# Skin Adhesive Tapes: An Effective Wound Closure Method for Percutaneous Vertebral Body Stenting

**DOI:** 10.7759/cureus.67859

**Published:** 2024-08-26

**Authors:** Cassie Yang, Theophilus Qiu, Reuben Soh Chee Cheong, Youheng Ou Yang

**Affiliations:** 1 Orthopaedic Surgery, Singapore General Hospital, Singapore, SGP; 2 Yong Loo Lin School of Medicine, National University Singapore, Singapore, SGP

**Keywords:** orthopedic spine surgery, surgery spine, skin adhesive tapes, wound healing, vertebral body stent

## Abstract

Background

Skin adhesive tapes (SATs) are hypoallergenic adhesive tapes commonly used for wound closure in percutaneous vertebroplasty (PVP). Vertebral body stenting (VBS) is a metallic balloon-expandable stent used to treat vertebral body fractures. Its balloon and stent deployment involves a larger stab incision and pedicle bore tract than PVP, increasing the risk of bleeding and wound complications. This study evaluated the outcome and complications of VBS wound closure with SAT and the reasons for conversion to conventional suture closure (SC).

Material and methods

A retrospective series of patients who underwent VBS from May 2019 to March 2021 were identified from review of computerized medical records. Data were collected for wound closure method, reason for SC, number of operative levels, postoperative wound complications of contact dermatitis, tension blisters, tape dislodgement, surgical site infection, wound dehiscence, symptomatic hematoma and return to operating theater. The wounds were assessed for complete healing and cosmesis at outpatient follow-up visits.

Results

A total of 36 patients were identified. SAT closure was performed in 33 (91.6%) patients, while SC was performed in three (8.3%) patients. Unplanned conversion to SC was required in two (5.5%) patients due to continued intraoperative wound bleeding, while one (2.7%) patient had planned SC as part of a staged operation. Uneventful closures occurred in 32 (97.0%) of SAT closures. One (3%) SAT closure patient developed postoperative blood-soaked dressings and tape dislodgement, requiring reapplication of the SATs at the ward with uneventful recovery thereafter. No patient with SAT closure developed contact dermatitis, tension blisters, surgical site infection, wound dehiscence, symptomatic hematoma, or required return to theater. All SAT closure patients had complete wound healing at outpatient follow-up at six weeks. No SAT closure was found to be cosmetically unacceptable or required wound revision for any reason at up to one year postoperatively.

Conclusion

SATs are a safe and reliable means of wound closure for VBS. Conversion to SC due to continued intraoperative wound site bleeding is rarely required.

## Introduction

Wound closure is a critical component of surgery, which influences the immediate postoperative healing process such as wound dehiscence, surgical site infection, hematoma formation, scarring, and patient comfort. Traditionally, suture closure (SC) has been relied upon as the gold standard for closing surgical wounds. SC provides a strong and reliable closure while also achieving local hemostasis. The use of SC is not without disadvantages. Sutures can cause additional trauma to the skin and underlying tissues by providing a nidus for bacterial colonization and subsequent surgical site infection [1] and cause tissue strangulation if improperly tensioned. These factors contribute to suboptimal cosmetic outcomes, including prominent scarring.

Skin adhesive tapes (SATs) are promising alternatives to sutures, having the capacity to provide mechanical and strangulation-free support for wounds. They are made of a hypoallergenic adhesive reinforced with polymer fibers, which allows them to adhere to and oppose the superficial wound edges with minimal tension, providing similar tensile strength as skin sutures while maintaining epidermal integrity [2]. For smaller surgical incisions, such as in arthroscopic wounds, SATs have been found to deliver lower complication rates than closure with nylon sutures [3,4]. Compared to conventional suture or staple closure, SATs deliver comparable [5] or better [6,7] cosmetic results and require less time for closure [8]. Limitations to SAT closure include long, deep, or gaping wounds or wounds located at areas of high movement such as those overlying joints. In these cases, SATs provide inadequate support and require additional subdermal sutures [9] to maintain closure integrity. Bleeding or oozy wounds are another relative contraindications to SAT closure, as the moisture results in adhesive failure, leading to a loss of tissue support. Complications associated with SATs are rare but include contact dermatitis, tension blisters, tape dislodgement, wound dehiscence, and wound infection [10].

Percutaneous vertebroplasty (PVP) is a well-established technique used to treat vertebral compression fractures arising from various etiologies, including osteoporosis, osteolytic metastasis, and multiple myeloma. PVP utilizes percutaneous cannulas introduced into the fractured vertebral body via small sub-centimeter stab incisions. Polymethyl methacrylate (PMMA) bone cement is injected through the cannulas to stabilize the fracture site and provide pain relief. These highly minimally invasive stab incisions have previously been successfully closed by SATs at our institution and others.

Vertebral body stenting (VBS), introduced in 2010 [11], is a refinement of PVP. VBS stentoplasty appears to be a similar procedure but has key procedural differences in addressing the drawbacks of PVP such as cement leaks caused by cement pressurization and large open vascular channels [12,13,14], residual vertebral body kyphosis [15]. In VBS stentoplasty, a metallic VBS stent is expanded and deployed by an inflatable balloon within the fractured vertebral body. The inflatable balloon creates a low-pressure void to reduce cement leaks [16,17], while the implanted VBS stent effectively maintains fracture reduction and reduces the loss of vertebral body height after the momentary absence of support prior to cementation [18].

As a tradeoff to these benefits, the VBS access kit requires a larger working sleeve of 4.7 mm in diameter as opposed to a 2.59 mm cannula in PVP. This 330% increase in surface area is necessary to accommodate both the metallic stent and inflatable balloon for deployment. In addition, the implantation of metal stents demands precise placement of the access kit compared to the larger and more forgiving target area for cement cannula placement in PVP. VBS stentoplasty thus requires larger skin incisions of 1.5-2cm, causes greater soft tissue trauma, and generates a bigger pedicle bore tract with exposed cancellous bone with the potential to continue bleeding. In addition, the thoracolumbar spine that is an area under tension and subject to friction when the patient is supine. These factors increase the risk of bleeding and wound complications, which may render SAT closure unsuitable.

This study aims to evaluate the outcomes and complications of SAT closure of VBS wounds as well as the rate and reason for conversion to SC closure. An early draft of this paper was previously posted to the preprint server "Research Square" on July 7, 2023.

## Materials and methods

Study design and setting

This study is a retrospective case series of patients who underwent VBS stentoplasty and subsequent wound closure from either SATs or SC from May 2019 to March 2021, based on a review of electronic medical records at a tertiary hospital in Singapore.

Participant eligibility criteria

The inclusion criteria were all patients who were operated on by either of two surgeons who practiced the following surgical technique for VBS stentoplasty and observed the described skin closure protocol and subsequent wound management.

Methodology and data collection

Surgical Technique for VBS Stentoplasty

**Figure 1:**
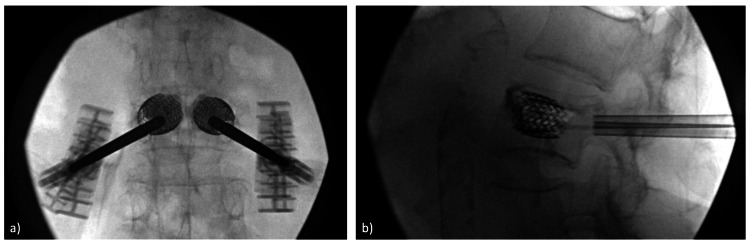
a) Intraoperative anterior-posterior view of vertebral body stenting (VBS) being inflated. b) Intraoperative lateral view of the expanded VBS prior to cement implantation.

Patients are sedated and positioned prone on a radiopaque surgical table. After identifying the levels of interest in intraoperative radiography, Marcaine and adrenaline are infiltrated along the planned cannula tracts and into the periosteum surrounding the pedicle entry site. Stab incisions of approximately 1.5-2 cm are made. A guidewire is inserted through the skin incisions, across the pedicles, and into the vertebral body, guided by sequential radiographs. A working sleeve is advanced over the guidewire, and the guidewire is removed. An access channel is drilled through this working sleeve, which is tamped down with a blunt plunger. The VBS stent size is templated according to the markings on the blunt plunger. The VBS stents and balloons are inserted via the working sleeve. They are subsequently inflated with contrast saline via a hand-held pump with an integrated pressure gauge. Balloon inflation with stent expansion is performed until there is adequate fracture reduction or if a pressure of 30 atmospheres and/or the maximum height of the metal stent is reached. Both balloons are then deflated and retrieved, leaving the expanded VBS stents to maintain the reduction (Figure 1). PMMA bone cement is subsequently injected through the working sleeve to reinforce the implant within the treated vertebral body.

Skin Closure Protocol and Subsequent Wound Management

**Figure 2:**
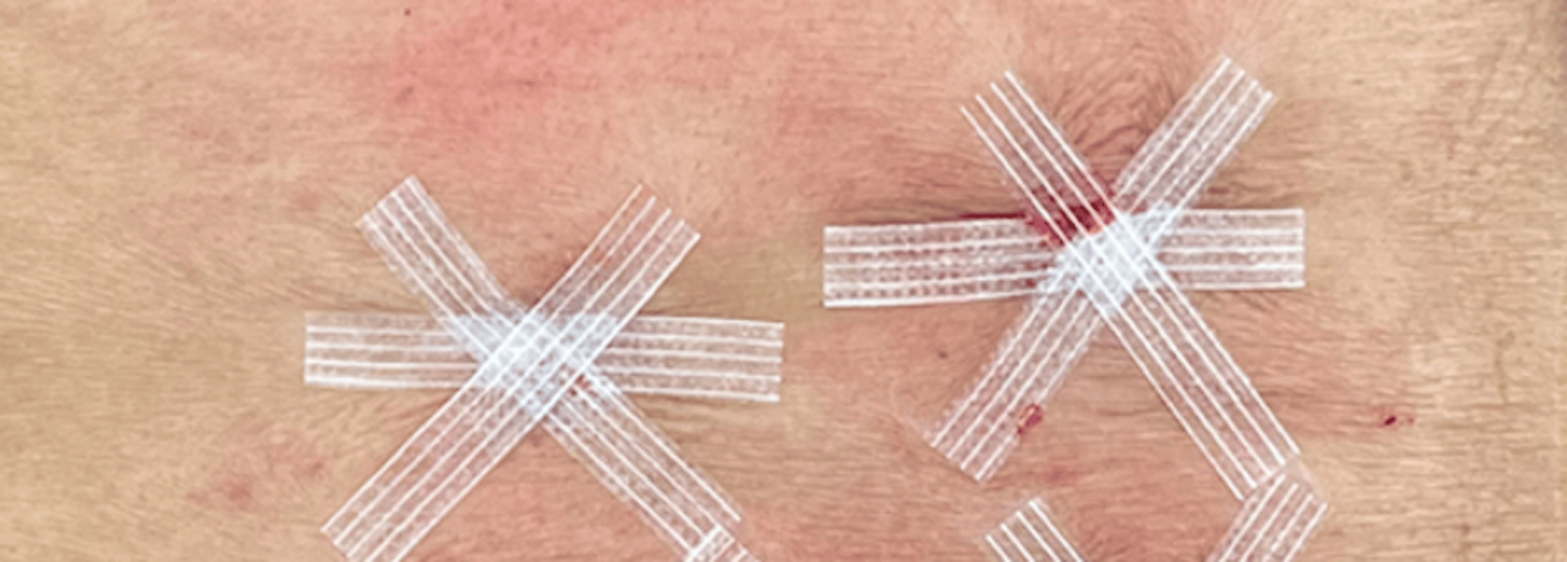
Skin adhesive tape closure of vertebral body stenting wounds in a stellate pattern. This configuration was chosen to create a multidirectional support to avoid dislodgement when patients shift in bed.

After the withdrawal of the VBS access kit, wounds were irrigated with normal saline. If remnant bleeding was noted from the wound, manual pressure was applied for approximately 30 seconds. In wounds without significant bleeding after irrigation and manual compression, three SATs measuring 6 × 50 mm (3M™ Steri-Strip™ (3M, St. Paul, MN, USA), 6 × 100 mm, cut to halves) were applied to each incision in an overlapping fashion (Figure 2).

If bleeding failed to stop after manual compression, SAT closure was abandoned, and SC was performed with monofilament simple interrupted sutures (Prolene™ 3/0, Ethicon, Raritan, NJ, USA), which successfully provided local hemostasis.

**Figure 3:**
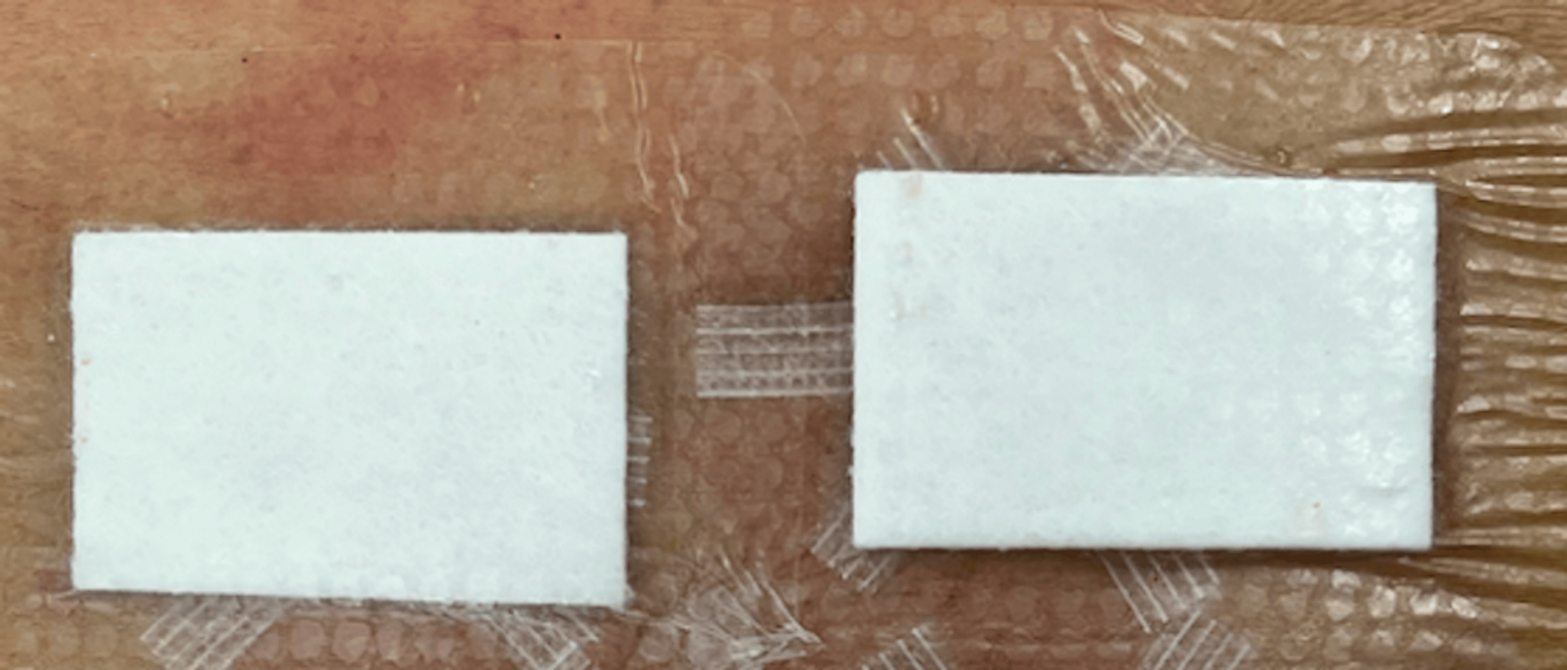
Non-adherent polyurethane dressing applied over skin adhesive tapes.

All wounds were dressed with a polyurethane film surgical dressing (Opsite Post-Op, Smith & Nephew, Hull, UK) (Figure 3).

The majority of the SATs were removed at the first dressing change, three days after surgery. Dry dressings were continued until sufficient epithelization occurred for the wound to be left exposed. This was typically within seven days and was decided by a general practitioner or nurse if the patient had been discharged outpatient or by the treating surgeon if the patient had remained inpatient for any reason. In cases with significant gaps in the wound after the first dressing change, SATs were reapplied until wound healing had progressed further. SC wounds required additional suture removal at approximately 14 days postoperatively.

Data Collection

Data were collected for patient demographics and biodata, number of operative levels, wound closure method, and postoperative skin-related wound complications: contact dermatitis, tape dislodgement, tension blisters, wound dehiscence, wound infection, postoperative bleeding, and return to operating theater. These patients were also screened for risk factors for poor postoperative outcomes, including wound site dehiscence and infection, as per described in the American College of Surgeons National Surgical Quality Improvement Program (ACS NSQIP®). The outcomes of the wounds were recorded at the patients’ outpatient clinic follow-up post-discharge at six weeks, three months, and one year. The wound was visually evaluated for complete healing or if there was unsightly scarring requiring wound revision. All patients were also asked if they had any concerns with the wound. Cosmetic outcomes were deemed satisfactory if patients did not raise any dissatisfaction or required wound revision for any reason.

The collection of data was obtained under Institutional Review Board waiver. An independent third party not otherwise involved in the study team performed the data extraction. The data were anonymized and keyed into an electronic Excel spreadsheet (Microsoft Corporation, Redmond, WA) prior to handover to the study team.

## Results

**Figure 4:**
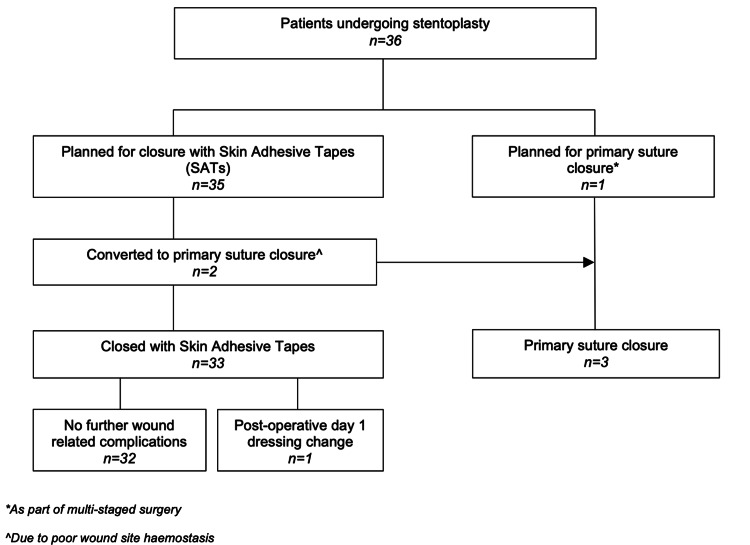
Flowchart of closure outcomes after transpedicular vertebral body stenting stentoplasty.

**Figure 5:**
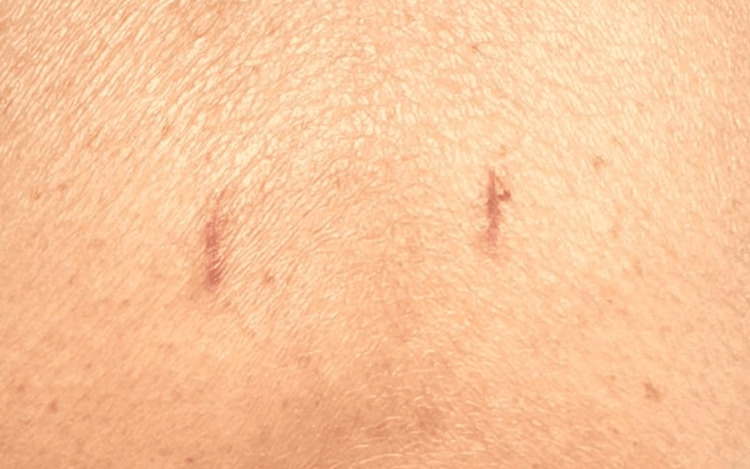
Typical appearance of post-vertebral body stenting wound closed by skin adhesive tape at six weeks.

VBS stentoplasty was performed in 36 patients with a total of 45 levels of VBS stentoplasty performed. SAT closure was performed in 33 (91.6%) patients, while conventional SC was performed in three (8.3%) patients. Unplanned conversion to SC was required in two (5.5%) patients due to intraoperative continued wound bleeding, while one (2.7%) patient had planned SC as part of a staged operation. Uneventful closures occurred in 32 (97.0%) of SAT closures. One (3%) SAT closure patient developed blood-soaked dressings and tape dislodgement at postoperative day 1 and required early reapplication of the SATs at the ward with uneventful recovery thereafter. No patient with SAT closure developed contact dermatitis, tension blisters, surgical site infection, wound dehiscence, symptomatic hematoma, or required return to theater (Figure 4). All SAT closure patients had complete wound healing at outpatient follow-up at six weeks (Figure 5). No SAT closure was found to be cosmetically unacceptable or required wound revision for any reason at up to one year postoperatively.

Within the patients who received SAT closure, 27 (81.8%) had at least one risk factor for wound dehiscence, including steroid use for chronic conditions (n = 4, 12.1%), disseminated cancer (n = 5, 15.1%), diabetes mellitus (n = 7, 21.1%), hypertension requiring medication (n = 18, 54.6%), and dialysis (n = 1, 3.03%). The complete demographics and biodata of the patients (Table 1) and risk factors for wound dehiscence (Table 2) are as detailed.

**Table 1:** Demographics and biodata of patients ASA, American Society of Anesthesiologists physical status classification system

Demographics	n	%
Gender		
Male	10	28
Female	26	72
Age		
≤50	3	8
51 to 60	7	19
61 to 70	5	14
71 to 80	12	33
81 to 90	8	22
≥90	1	3
Ethnicity		
Chinese	30	83
Malay	4	11
Indian	1	3
Others	1	3
BMI		
<18.5	5	14
18.5 to 24.9	19	53
25 to 29.9	6	17
≥30	6	16
ASA		
I	0	0
II	20	56
III	16	44
IV	0	0

**Table 2:** Risk factors for poor postoperative outcomes as per the American College of Surgeons National Surgical Quality Improvement Program (ACS NSQIP®) CHF, congestive heart failure; COPD, chronic obstructive pulmonary disease; SAT, skin adhesive tape

Risk factors	Whole sample, n = 36		SAT, n = 33		Primary closure, n = 3	
	n	%	n	%	n	%
Steroid use for chronic conditions	4	11.11	4	12.12	-	-
Ascites <30 days before surgery	-	-	-	-	-	-
Systemic sepsis <48 hours prior to surgery	-	-	-	-	-	-
Ventilator dependent	-	-	-	-	-	-
Disseminated cancer	6	16.67	5	15.15	1	33.33
Diabetes	7	19.44	7	21.21	-	-
Hypertension requiring medication	18	50.00	18	54.55	-	-
CHF <30 days prior to surgery	-	-	-	-	-	-
Dyspnea	-	-	-	-	-	-
Current smoker <1 year prior to surgery	-	-	-	-	-	-
History of severe COPD	-	-	-	-	-	-
Dialysis	1	2.78	1	3.03	-	-
Acute renal failure	-	-	-	-	-	-

For patients with unplanned conversion to SC, both patients had ongoing bleeding at the wound site at the end of the procedure. This was attributed to underlying coagulopathy, as one patient had perioperative aspirin use while the other had severe thrombocytopenia due to underlying bone marrow disease.

The patient with a planned SC closure had VBS stentoplasty performed as part of a staged procedure. SC was performed to immediately secure the wound to accommodate an intra-theater positional change from prone to supine on a traction table for a femoral intramedullary nail insertion.

## Discussion

This is the first study to critically evaluate the use of SATs in the closure of VBS stentoplasty wounds. VBS stentoplasty bears similarities to PVP but requires larger wounds (50-100% longer), creates more tissue trauma, and has larger exposed cancellous bone with bleeding. A large majority of patients, 91.6% (n = 33), were able to receive SAT closure without complications and with acceptable cosmetic results. The study cohort represented a wide range of patient demographics, including the elderly, with 25% of the cohort above the age of 80 (n = 9), and large body habitus, with 34% (n = 12) of the cohort found to have a BMI of >25, with a further 16% (n = 6) > 30. A BMI > 30 is indicative of clinical obesity. Elderly patients often have loose and frail skin, while obese patients have deep wound tracts and large amounts of subcutaneous fat to support. These local anatomical factors potentially render SAT closure problematic due to the concern of inadequate wound closure support derived solely from the adhesive tension on the skin. Both groups of patients are also at risk of tension blisters due to poor skin conditions and the amount of skin support required. In addition, the study cohort had a high rate of comorbid conditions known to affect wound healing and wound dehiscence, with 81.8% (n = 27) of patients having the presence of at least one such factor according to the criteria selected by ACS NSQIP®. Patients of ethnicities with darker skin pigmentation (Indian and Malay) 15% (n = 6) are at higher risk of keloid formation [19], yet no patient complained of unsightly wounds. As such, the authors believe that the results of this study are generalizable to most patients.

While VBS stentoplasty may be closed by SATs, not all patients were suitable. In cases where VBS stentoplasty was performed as part of a staged operation, immediate wound closure security with SC was required in 2.7% (n = 1) of patients. This allowed the patient to be repositioned intraoperatively and tension applied to the limbs. Bleeding and oozy wounds also introduce moisture at the site of skin adhesion, leading to adhesive failure and early SAT dislodgement. Unplanned conversion to SC was observed in 5.7% (n = 2) of patients due to ongoing bleeding. In this study, these patients had risk factors for coagulopathy. In cases of failed SAT closure, SC closure successfully provided additional wound support and hemostasis, thus affirming the role of traditional SC closure in VBS stentoplasty. Following intraoperative hemostasis as described in our VBS stentoplasty and SAT closure protocol, 3% (n = 1) of patients had blood-soaked wounds on postoperative day 1 with tape dislodgement. This could be due to inadequate ongoing hemostasis achieved solely from superficial skin apposition. However, this was rectified easily in the ward by removal of the soaked SATs and immediate reapplication, avoiding a return to the operating theater or conversion to primary sutures for wound closure. The authors thus advise caution on the use of SATs in patients with bleeding diathesis, such as a background of malignancy, anticoagulant use, or liver disease. As the decision to proceed with SAT closure is still guided by clinical considerations, patients with extremely frail skin, severe obesity, or who require immediate wound closure security may not be suitable.

The benefits of SATs are manifold. Compared to skin sutures and staples, SATs are an easy-to-apply and non-invasive method of skin closure. SATs eliminate the risk of introducing skin infections via the suture needle or suture material and avoid scarring associated with suture/staple entry and exit sites and wound strangulation. This was evidenced by the lack of any surgical site infections, wound dehiscence, or unsatisfactory cosmetic outcomes. SATs are faster to apply than conventional skin sutures and have been found to save 1.5 to 3.5 minutes of operating time per patient compared to skin sutures [20]; in high-volume centers, the overall time saved may be significant in facilitating the adding on of another case to the list. Otherwise, such time-saving may be reallocated to other activities for the operating theater staff, such as cleaning and turnaround time, contributing to reduced stress and improved clinical quality. As SATS do not require the removal of stitches or staples, the patients also experienced reduced outpatient time and cost [21].

A limitation of this study is its nature as a retrospective case series with small sample size, with data collected from only a single institution and the lack of a control group. Further studies to compare wound outcomes between SAT or SC closure in VBS stentoplasty are recommended, especially with regards to its use in patients with bleeding diathesis, patients with preexisting skin conditions, extreme obesity, or old age to expand the evidence base for use in these patients.

## Conclusions

SATs are a simple, safe, and effective means of wound closure for the majority of percutaneous VBS procedures with acceptable cosmetic outcomes. Potential benefits include saving operative time, eliminating peri-incisional puncture wounds from sutures or staples, puncture site bacterial ingress, and foreign material within the wound. SAT closure also provides cost and time savings from avoiding future outpatient sutures or staple removal. Conversion to conventional suture or staple closure is required if homeostasis cannot be achieved by local pressure at the wound site or if a more secure closure is required immediately as part of a staged procedure.
